# Red and green colors emitting spherical-shaped calcium molybdate nanophosphors for enhanced latent fingerprint detection

**DOI:** 10.1038/s41598-017-11692-1

**Published:** 2017-09-14

**Authors:** L. Krishna Bharat, G. Seeta Rama Raju, Jae Su Yu

**Affiliations:** 1 Department of Electronic Engineering, Institute for Wearable Convergence Electronics, Kyung Hee University, Yongin-si, Gyeonggi-do 17104 Republic of Korea; 2 Department of Energy and Materials Engineering, Dongguk University, Seoul, 04620 Republic of Korea

## Abstract

We report the synthesis of spherical-shaped rare-earth (Eu^3+^ and Tb^3+^) ions doped CaMoO_4_ nanoparticles in double solvents (IPA and H_2_O) with the help of autoclave. The X-ray diffraction patterns well match with the standard values and confirm the crystallization in a tetragonal phase with an I4_1_/a (88) space group. The luminescence spectra exhibit the strong red and green emissions from Eu^3+^ and Tb^3+^ ions doped samples, respectively. The X-ray photoelectron spectroscopy results show the oxidation states of all the elements present in the sample. The temperature-dependent luminescence spectra reveal the stability of Eu^3+^ and Tb^3+^ ions doped samples. The red- and green-emitting Eu^3+^ and Tb^3+^ ions doped CaMoO_4_ samples were used for detection and enhancement of latent fingerprints which are the common evidences found at crime scenes. The enhanced latent fingerprints obtained on different surfaces have high contrast with low background interference. The minute details of the fingerprint which are useful for individualization are clearly observed with the help of these nanopowders.

## Introduction

Nanoscience and nanotechnology evoke the research interest in synthesizing materials owing to their huge potential for applications in wide range of fields^1^. The self-assembled hierarchical structures having particular shapes and novel properties are important in the area of material synthesis and its application^2, 3^. They can bridge the nanoscale and microscale world and provide promising functions. Such features urge and initiate scientists in synthesizing inorganic hierarchical structures assembled by nanoparticles. This area of research has also got much attention and has an impact on forensic science for detecting latent fingerprints on potentially touched items^4, 5^. Latent fingerprints are not visible to the naked eye and need enhancement for identification and visualization. Among many identification techniques used for fingerprint detection, powdering is one technique which has been used since the early days of detection^6^. As we know, there are three main kinds of powders used for fingerprint detection i.e., standard, metallic and luminescent powders. The standard powder consists of adhesive polymer with a stain for contrast, and the metallic powder corresponds to powders of metals like Ag, Au and Pb^7^. These traditional powders have some difficulties and are unsafe to the human health and environment. The unique way to subdue such constrains is the use of rare-earth ions doped luminescent materials.

Rare-earth ions doped materials have characteristic properties and are applied in various fields ranging from electronics to biology and so on^8–10^. However, different types of host materials were studied and oxide-based materials were of significant interest because of their lower toxicity and robustness^11^. Metal molybdates of general formula AMoO_4_ (A = Ca, Sr, Ba) are one of the oxide-based host materials which give excellent luminescent properties due to the property of energy transfer from host lattice to the dopant ions^12, 13^. Among these metal molybdates, CaMoO_4_ is one of the host materials which belong to the scheelite family having tetragonal structure with a space group of I41/a. This material is highly transparent and allows a large range of light to pass through without weakening in luminescence. Additionally, these materials possess good chemical and physical properties in comparison to other oxide materials.

Many researchers have focused on and reported the synthesis of controlled CaMoO_4_ morphologies by following novel synthesis techniques. Different morphologies i.e., flower-like, doughnut-like, peanut-like, erythrocyte-like, walnut-like structures and so on were synthesized^14–17^. Recently, Liu *et al*. have demonstrated the controlled synthesis of CaMoO_4_ microspheres with PDDA^18^. The preparation of CaMoO_4_ takes a long synthesis time of 12 h and the particle size varies in the range of 1-2 μm. In this work, we demonstrate a relatively fast synthesis of spherical-shaped nanostructures formed with nano building blocks. The structural and optical properties were studied and the nanopowders were used to efficiently detect the latent fingerprints on different surfaces.

## Results and Discussion

**Figure 1:**
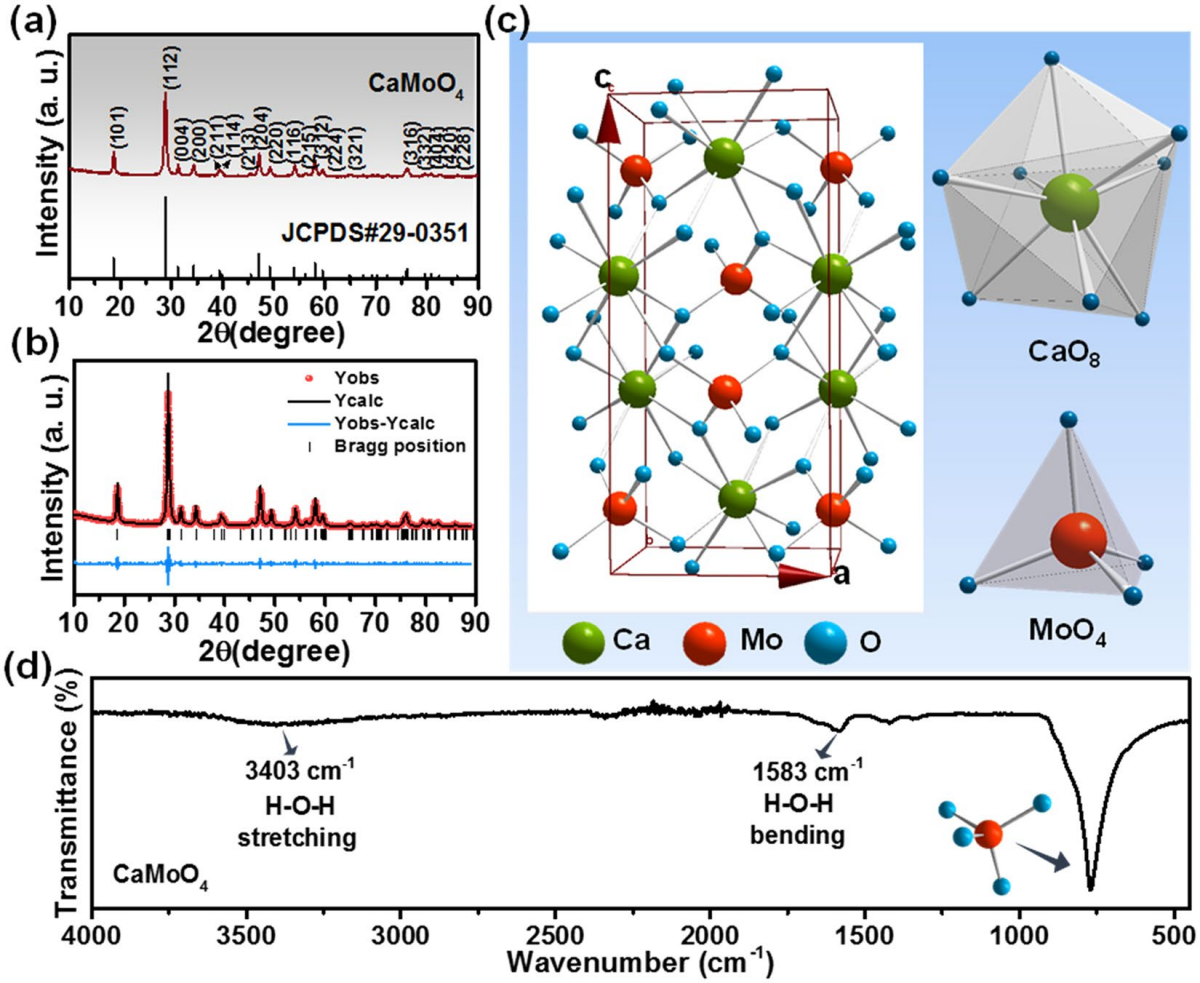
(**a**) XRD pattern, (**b**) XRD refinement, (**c**) crystal structure and (**d**) FTIR spectrum of CaMoO_4_.

Figure 1(a) shows the XRD pattern of CaMoO_4_ sample along with the standard JCPDS #29-0351. The XRD data well matched with the standard values, the CaMoO_4_ sample was crystallized in tetragonal phase with a space group of I4_1_/a (88) and no impurity peaks were found. To support the above mentioned statement, Rietveld refinement was performed on the XRD pattern using the FULLPROF software. The refined pattern with the observed (red), calculated (black), difference (blue) patterns and bragg-positions is shown in Fig. 1(b). The refinement parameters and the calculated and experimental cell parameters are tabulated as shown in Table 1. The lattice parameters obtained for CaMoO_4_ are a = b = 5.229 Å, c = 11.439 Å and V = 312.796 Å^3^. The CaMoO_4_ scheelite structure unit cell is shown in Fig. 1(c). The tetragonal structure with body centered inversion symmetry has Ca and Mo sites with S_4_ point symmetry. The crystal structure has CaO_8_ polyhedra and MoO_4_ tetrahedra units and the CaO_8_ unit shares four of its edges with four other polyhedrons through oxygen atoms. The MoO_4_ tetrahedra and CaO_8_ polyhedra are also connected through oxygen atoms and each oxygen atom is linked with one Mo atom and two Ca atoms^19^. The CaO_8_ units have two different Ca-O bond lengths (2.429 and 2.442 Å) and the MoO_4_ units have one Mo-O bond length (1.810 Å). The individual CaO_8_ and MoO_4_ units are shown in Fig. 1(c). Figure 1(d) shows the FTIR spectrum of CaMoO_4_ sample in the wavenumber range of 4000-450 cm^−1^. The spectrum consists of the bands at 3403 and 1583 cm^−1^ and the characteristic band of molybdate below 1000 cm^−1^. The bands at 3403 and 1583 cm^−1^ are due to the water molecules absorbed on to the sample and correspond to the H-O-H stretching and H-O-H bending vibrations, respectively^20^. The band centered at 770 cm^−1^ corresponds to the anti-symmetric stretching vibrations (ν_3_) of MoO_4_ tetragonal clusters^21^.

**Table 1:** Calculated and experimental data and refinement parameters of CaMoO_4_.

	Calculated	Experimental
a = b (Å)	5.2291	5.2246
c (Å)	11.4394	11.4349
Angles (α, β, γ)	(90, 90, 90)	(90, 90, 90)
Atomic Positions Ca(4b) (x, y, z) Mo(4a) (x, y, z) O (16 f) (x, y, z)	(0, 1/4, 5/8) (0, 1/4, 1/8) (0.1537, −0.0004, 0.2087)	(0, 1/4, 5/8) (0, 1/4, 1/8) (0.1507, 0.0086, 0.2106)
Volume (Å^3^)	312.799	
Refinement Parameters	R_p_ = 4.58	
	R_wp_ = 5.78	
	R_exp_ = 3.83	
	χ^2^ = 2.35	

The CaMoO_4_ spherical nanoparticles were synthesized by mixing appropriate amounts of calcium nitrate tetrahydrate, ammonium heptamolybdate tetrahydrate, sodium hydroxide and EDTA in IPA and DI water mixture as mentioned in the experimental section. The possible reaction mechanism for the formation of CaMoO_4_ is as follows:

1 $${{\rm{Ca}}}^{2+}{+\mathrm{2OH}}^{-}\to {\rm{Ca}}{({\rm{{\rm O}}}{\rm{{\rm H}}})}_{{\rm{2}}}$$

2 $${{\rm{Mo}}}_{{\rm{7}}}{{{\rm{O}}}_{{\rm{24}}}}^{6-}+{{\rm{4H}}}_{{\rm{2}}}{\rm{O}}\to {{{\rm{7MoO}}}_{{\rm{4}}}}^{2-}+{{\rm{8H}}}^{+}$$

3 $${\rm{7Ca}}{({\rm{{\rm O}}}{\rm{{\rm H}}})}_{{\rm{2}}}+{{\rm{7MoO}}}_{{\rm{4}}}^{2-}+{{\rm{14H}}}^{+}\to {{\rm{7CaMoO}}}_{{\rm{4}}}+{{\rm{14H}}}_{{\rm{2}}}{\rm{O}}$$

When the solution-II was mixed with the solution-I, as described in the experimental section, the OH^−^ anions from the NaOH solution react with the Ca^2+^ cations and form the Ca(OH)_2_. The formation of Ca(OH)_2_ is also confirmed by taking the XRD and it matches well with the JCPDS #72-0156 (Fig. S1). The heptamolybdate dissolved in DI water gives MoO_4_^2−^ monomeric oxyanion which is similar to the earlier reports^22^. The solution containing these monomeric oxyanions was added drop by drop into the solution containing Ca(OH)_2_, which further reacts and gives the final product CaMoO_4_ as shown in Eq. (3). The CaMoO_4_ synthesized in pure DI water rather gives nanoflake-like morphology as shown in Fig. S2. This is due to the ammonia-induced fast reaction between the calcium nitrate and ammonium heptamolybdate in DI water^23^. However, when IPA was added to the system, the reaction slows down and smaller particles are formed as shown in Fig. S3(a). Hence, it can be said that IPA plays a key role in obtaining the spherical-shaped CaMoO_4_ particles. These smaller particles aggregate and assemble into long spindle-shaped structures due to the presence of EDTA (inset of Fig. S3(a)) and are further formed into small clusters. These clusters grow further as the reaction time increases as shown in Fig. S3(b) and (c) and the Ostwald’s ripening also takes place, which results in the formation of bigger particles. Finally, the spherical-shaped CaMoO_4_ nanoparticles were obtained as shown in Fig. S3(d) due to the continuous assembly and Ostwald’s ripening processes.

**Figure 2:**
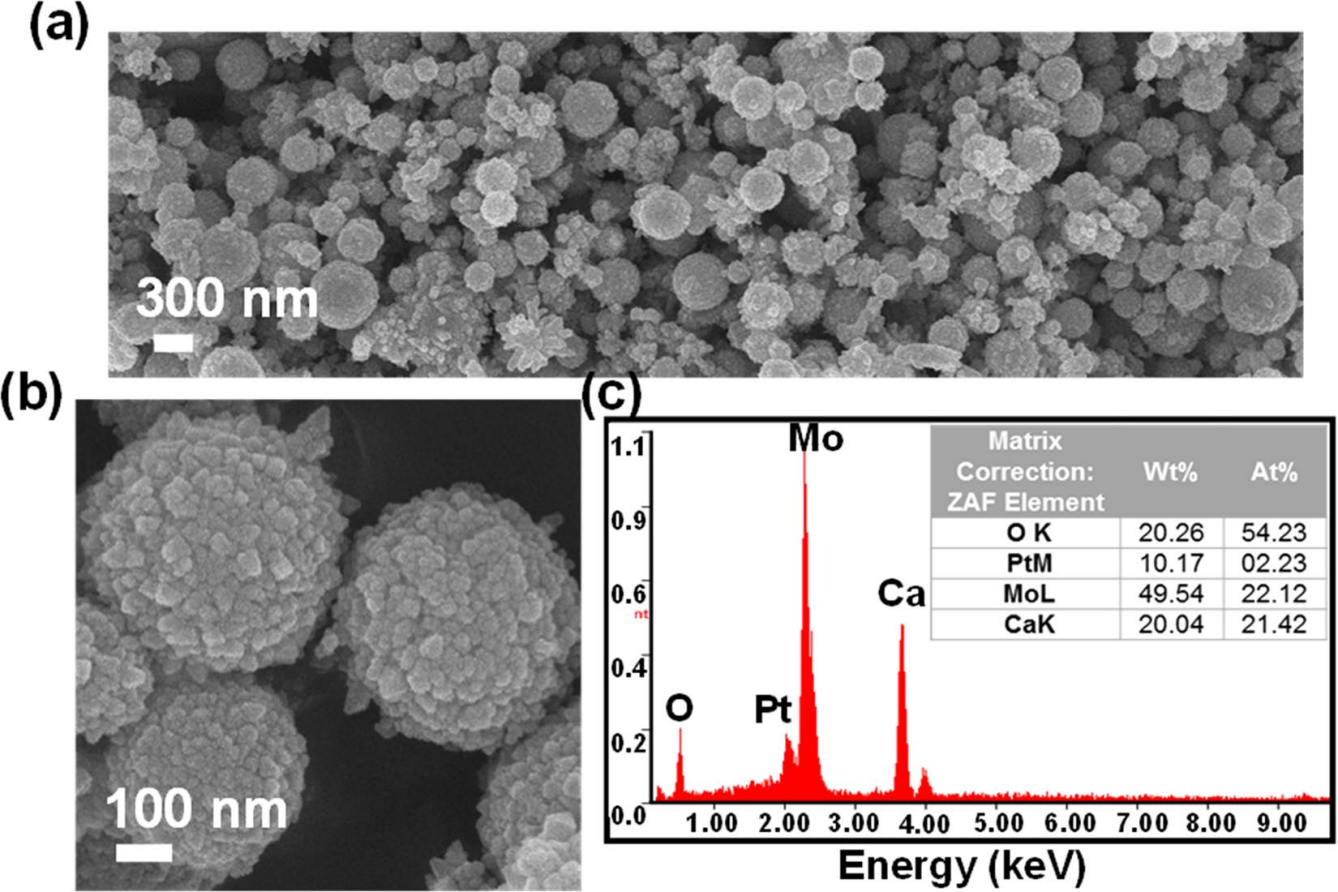
(**a**) Low-magnification FE-SEM image, (**b**) high-magnification FE-SEM image and (**c**) EDX spectrum (inset shows the elemental distribution table) of CaMoO_4_ sample.

Figure 2(a) shows the low-magnification FE-SEM image of the as-prepared CaMoO_4_ sample. From the FE-SEM image, it was observable that the formed particles are spherical in shape with sizes in the range of 300–500 nm. In Fig. 2(b), the high-magnification FE-SEM image revealed the rough surface on the spherical particles. Also, the spherical particles consist of smaller nanoparticles which are the building blocks. The chemical composition and atomic percentage distribution of elements were determined with the EDX spectrum, as shown in Fig. 2(c). The spectrum exhibited all the elements i.e., Ca, Mo and O and a small peak at approximately 2 keV which belongs to platinum (Pt) was observed. The presence is obvious due to the Pt coating done before measuring the sample. The atomic and weight percentage distribution of elements is tabulated and is shown in the inset of Fig. 2(c).

**Figure 3:**
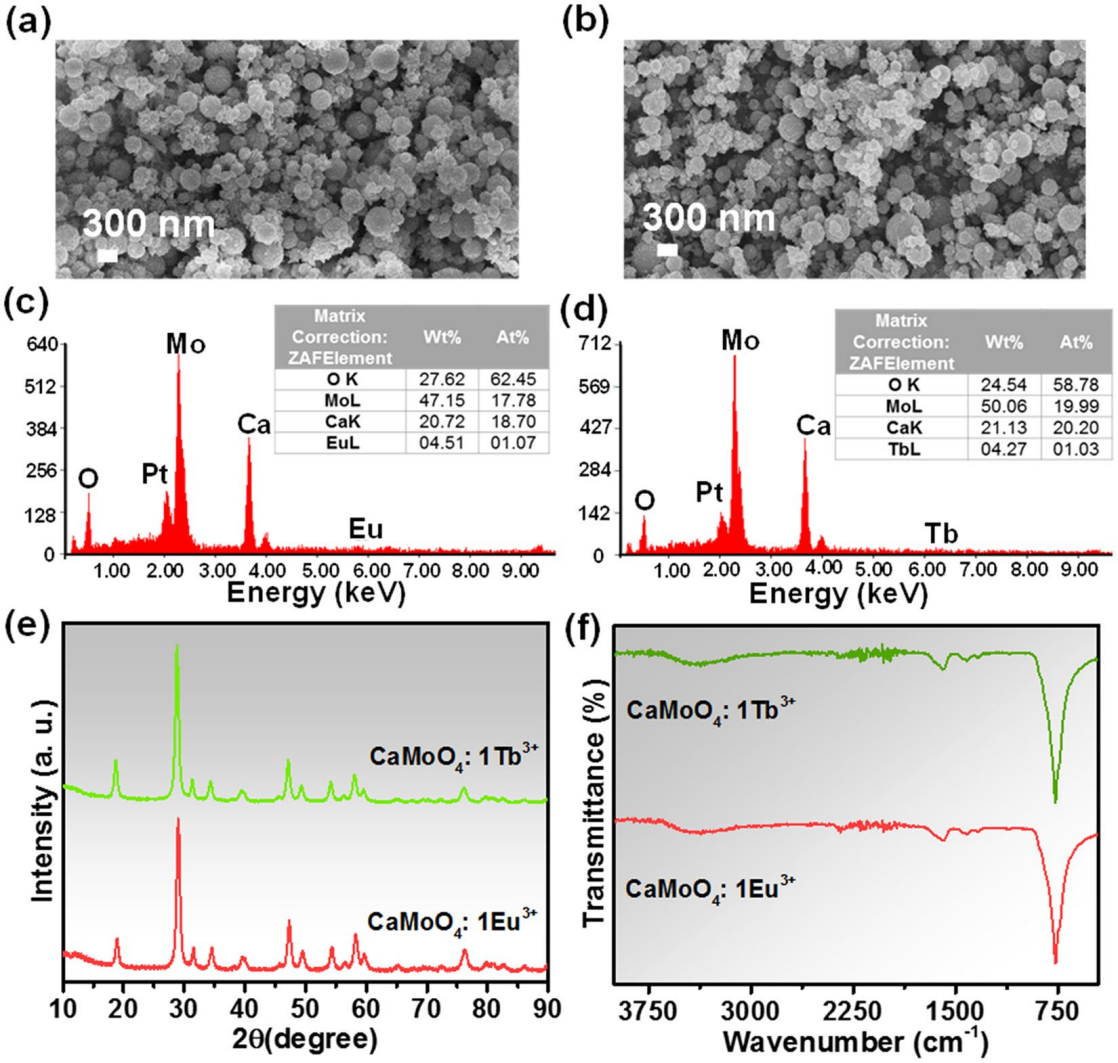
(**a**,**b**) FE-SEM images, (**c**,**d**) EDX spectra, (**e**) XRD patterns and (**f**) FTIR spectra of the Eu^3+^ and Tb^3+^ ions doped CaMoO_4_ samples.

Figure 3 shows the FE-SEM images, EDX patterns, XRD patterns and FTIR spectra of 1 mmol Eu^3+^ and 1 mmol Tb^3+^ ions doped CaMoO_4_ samples (hereafter referred as CaMoO_4_: 1Eu^3+^ and CaMoO_4_: 1Tb^3+^). The FE-SEM images in Fig. 3(a) and (b) displayed that the particles are spherical in shape and the doping of rare-earth elements does not have a great effect on the morphology of the CaMoO_4_ particles. As shown in Fig. 3(c) and (d), the EDX spectra revealed the elements present in Eu^3+^ and Tb^3+^ ions doped CaMoO_4_ samples. The spectra show all the elements i.e., Ca, Mo, O, Eu and Tb that are present in the samples. The insets show the table containing atomic and weight percentage distributions of elements. The XRD patterns of Eu^3+^ and Tb^3+^ ions doped CaMoO_4_ samples are shown in Fig. 3(e). The XRD patterns were well consistent with the standard JCPDS card value. Also, these XRD patterns well matched with that of the as-prepared CaMoO_4_ sample. With the doping of rare-earth elements, there are no significant changes in the XRD patterns, indicating that the Eu^3+^ and Tb^3+^ ions were completely doped into the Ca^2+^ sites. The difference in the ionic radii of Ca^2+^ and rare-earth ions of Eu^3+^ and Tb^3+^ was also very small (i.e., less than 30%) and very dilute concentrations were used in this experiment. Similarly, the FTIR spectra were also recorded for Eu^3+^ and Tb^3+^ ions doped CaMoO_4_ samples and they also matched well with that of the as-prepared CaMoO_4_ sample. The bending and stretching vibrations of H-O-H and H-O-H due to water molecules and the characteristic bands of molybdate were seen in the FTIR spectra.

**Figure 4:**
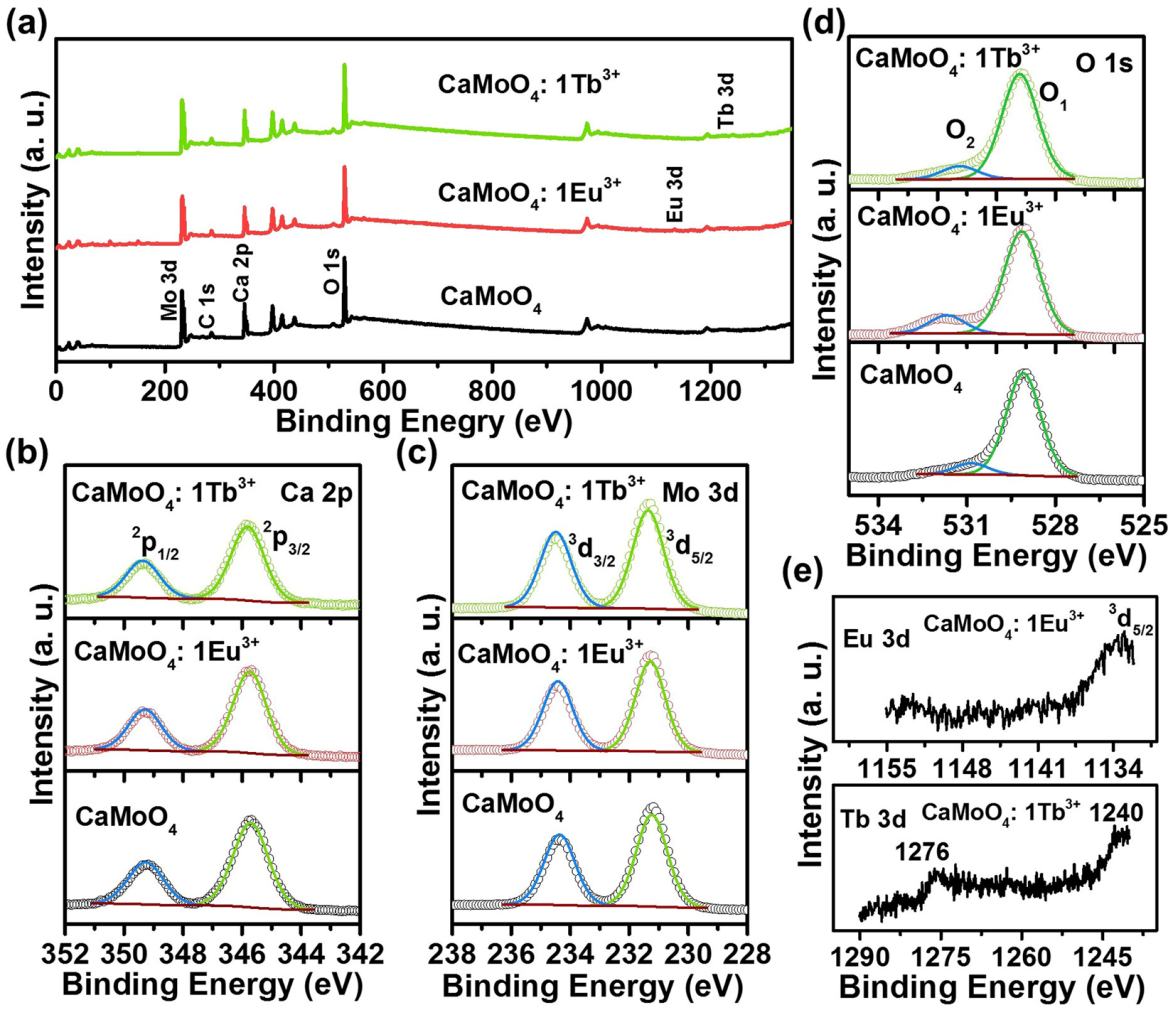
(**a**) Survey scan spectra, and high-resolution scan of (**b**) Ca 2p, (**c**) Mo 3d and (**d**) O 1 s for the undoped and doped CaMoO_4_ samples. High-resolution scan of (**e**) Eu 3d and (**f**) Tb 3d.

The oxidation states of the elements present in the molybdate samples were determined by a very sensitive technique i.e., XPS. The samples for XPS measurement were prepared by the following procedure; firstly, the powder samples were dispersed in DI water, drop-casted onto the cleaned silica substrate, dried and then used for measurements. The survey scan XPS spectra of the undoped and Eu^3+^ and Tb^3+^ ions doped CaMoO_4_ samples are shown in Fig. 4(a). The survey scan spectrum of CaMoO_4_ shows the peaks related to Ca, Mo and O and the doped samples also show similar peaks along with the Eu and Tb peaks for respective samples. The carbon 1 s peak in the spectra was due to the presence of trace amount of hydrocarbons during the XPS measurement. The high-resolution Ca 2p scan for CaMoO_4_ samples is shown in Fig. 4(b). As shown in Fig. 4(b), the two bands at binding energy (BE) values of 345.71 and 349.26 eV correspond to the Ca 2p_3/2_ and Ca 2p_1/2_, respectively. The full width at half maximum (FWHM) values for the two peaks were 1.41 and 1.41 eV and the area ratio, i.e., area of 2p_3/2_/area of 2p_1/2_ (A_Ca_) was 2.00 for CaMoO_4_ sample. When the Eu^3+^ and Tb^3+^ ions were doped, the BE values of 2p_3/2_ and 2p_1/2_ did not show significant changes (Fig. 4(b)), and the A_Ca_ values also remained the same i.e., 2.00. The FWHM values slightly decreased as shown in Table 2. From the above obtained results and BE values, it is believed that the calcium is stabilized in +2 oxidation state in the undoped and doped samples. Figure 4(c) shows the high-resolution Mo 3d scan for all the CaMoO_4_ samples. The Mo 3d scan of CaMoO_4_ revealed two peaks at BE values of 231.24 and 234.37 eV corresponding to the 3d_5/2_ and 3d_3/2_. The A_Mo_ (area of 3d_5/2_/area of 3d_3/2_) value was found to be 1.29 and the FWHM values were found to be 1.24 and 1.24 eV, respectively. For Eu^3+^ and Tb^3+^ ions doped samples, the BE values of 3d_5/2_ and 3d_3/2_ did not show significant changes and the A_Mo_ values also remained almost the same. The FWHM value of the Eu^3+^ ions doped sample decreased and for the Tb^3+^ ions doped sample, the FWHM value remained the same, which can be seen in Table 2. The results indicate that the molybdenum is stabilized in +6 oxidation state in the undoped and doped samples. Figure 4(d) shows the high-resolution O 1 s spectra of the undoped and doped samples. The O 1 s spectrum of CaMoO_4_ exhibited a peak around 529 eV which is slightly asymmetric and two symmetric Gaussian peaks were fitted and labeled as O_1_ and O_2_. The O_1_ peak is attributed to the lattice oxygen of the CaMoO_4_ host lattice. The O_2_ peak is attributed to the chemisorbed oxygen or hydroxyl group as mentioned in earlier reports^24^. In spite of that, the O_2_ peak at the higher BE is normally due to the oxygen vacancies present in the sample, which was in consistent with previous reports^25^. The A_O_ (O_2_/O_1_) values of the undoped and Eu^3+^ and Tb^3+^ ions doped samples were found to be 0.109, 0.176, and 0.124, respectively. The A_O_ value of the undoped sample is smaller than those of the doped samples, suggesting the enrichment of oxygen vacancies. This result is reasonable as the defect and oxygen vacancy concentration increases with the doping of trivalent ions into the divalent calcium sites. Figure 4(e) shows the high-resolution Eu 3d and Tb 3d spectra of the Eu^3+^ and Tb^3+^ ions doped CaMoO_4_ samples, respectively. The Eu 3d spectrum exhibited a peak around BE value of 1134 eV which corresponds to the 3d_5/2_. This is consistent with the reported values and indicates that the europium ions exist in +3 oxidation state^26^. Similarly, the Tb 3d spectrum showed two peaks around BE values of 1240 and 1276 eV which are in accord with the reported values, indicating that the terbium ions exist in +3 oxidation state^27, 28^.

**Table 2:** XPS results of the undoped and doped CaMoO_4_ samples.

Compound			BE (eV)	FWHM	Area	At %
CaMoO_4_	Ca 2p	Ca 2p_3/2_	345.71	1.41	37911.21	28.24
		Ca 2p_1/2_	349.26	1.41	18955.6	
	Mo 3d	Mo 3d_5/2_	231.24	1.24	60482.96	27.16
		Mo 3d_3/2_	234.37	1.24	46525.36	
	O 1 s	O1	529.09	1.37	79394.79	44.60
		O2	530.86	1.37	8687.48	
CaMoO_4_: Eu^3+^	Ca 2p	Ca 2p_3/2_	345.75	1.32	30490.1	25.90
		Ca 2p_1/2_	349.29	1.32	15245	
	Mo 3d	Mo 3d_5/2_	231.29	1.19	53272.5	26.47
		Mo 3d_3/2_	234.42	1.19	40978.8	
	O 1 s	O1	529.14	1.43	72910.47	47.52
		O2	531.67	1.43	12827.56	
CaMoO_4_: Tb^3+^	Ca 2p	Ca 2p_3/2_	345.81	1.33	34814.8	26.10
		Ca 2p_1/2_	349.36	1.33	17407.4	
	Mo 3d	Mo 3d_5/2_	231.36	1.24	60603.5	27.38
		Mo 3d_3/2_	234.49	1.24	46618.1	
	O 1 s	O1	529.21	1.44	85480.57	46.45
		O2	531.25	1.44	10608.43	

**Figure 5:**
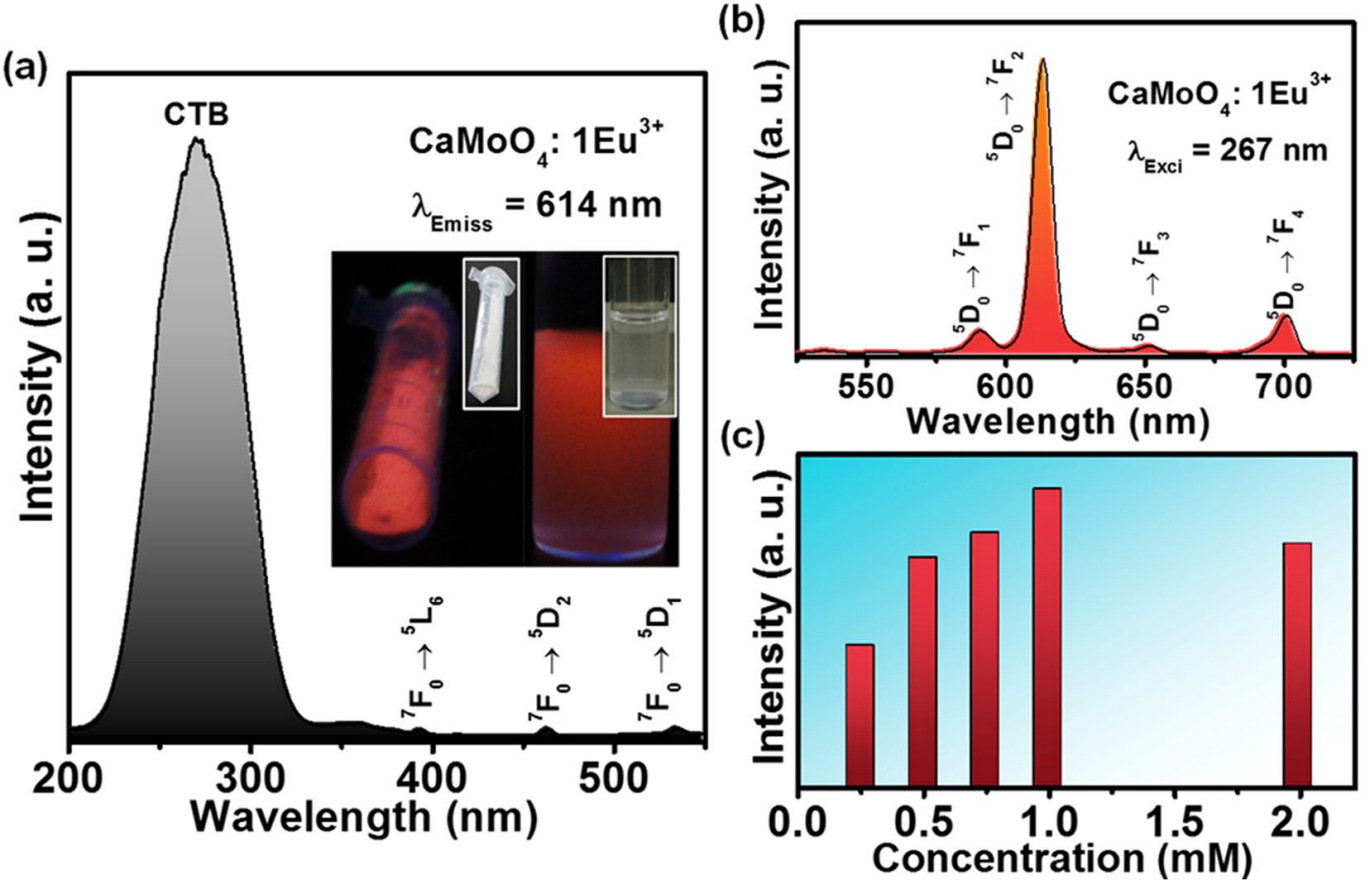
(**a**) PLE spectrum (inset shows the photographs of the powder sample and the powder dispersed in DI water under normal and UV lights), (**b**) PL emission spectrum and (**c**) PL emission intensity versus Eu^3+^ ion concentration for the Eu^3+^ ions doped CaMoO_4_ sample.

Figure 5(a) shows the photoluminescence (PL) excitation (PLE) spectrum of the Eu^3+^ ions doped CaMoO_4_ sample. The PLE spectrum was obtained by monitoring at an emission wavelength of 614 nm. The excitation spectrum revealed a broad band in the ultraviolet (UV) region and small f-f intraconfigurational transitions of Eu^3+^ ions in the longer wavelength region. The broad band centered at 267 nm is due to the charge transfer transition between the O^2−^ and Mo^6+^ and so it is named as charge transfer band (CTB). The presence of intense CTB also indicates that there will be an energy transfer process from MoO_4_^2−^ moieties to Eu^3+^ ions. The weak bands observed at 394, 464 and 534 nm are assigned to the ^7^F_0_ → ^5^L_6_, ^7^F_0_ → ^5^D_2_ and ^7^F_0_ → ^5^D_1_ electronic transitions of Eu^3+^ ions, respectively. Figure 5(b) shows the PL emission spectrum of the Eu^3+^ ions doped CaMoO_4_ sample. The emission spectrum was obtained in the wavelength range of 500–725 nm by monitoring at an excitation wavelength of 267 nm. The PL emission spectrum showed an intense band centered at 614 nm which is due to the hypersensitive electric dipole ^5^D_0_ → ^7^F_2_ transition. The other bands centered at 591, 652 and 700 nm are due to the transition from the high energy ^5^D_0_ level to the ground states ^7^F_1_, ^7^F_3_ and ^7^F_4_, respectively. The asymmetric ratio, i.e., ratio of intensities of ^5^D_0_ → ^7^ F_2_/^5^D_0_ → ^7^F_1_ transitions, is higher than 1. This indicates that the Eu^3+^ ions occupy the non-inversion symmetry site and exhibit red emission. In general, the Ca^2+^ ions in the host lattice occupy inversion symmetry site, so the Eu^3+^ ions doped into the Ca^2+^ sites should give the asymmetric ratio less than 1. The opposite was observed due to the polarization effect of MoO_4_^2−^ moieties present in the host lattice and it also affects the luminescence of the sample. The MoO_4_^2−^ moieties of the host lattice are useful to get strong emission from activator ions by transferring the absorbed excitation energy non-radiatively to the Eu^3+^ ions^12^. The emission colors from the powder sample and the powder dispersed in DI water were snapped under normal and UV lights and are shown in the inset of Fig. 5(a). The PL emission intensity increased with the increase in the dopant concentration and reached its maximum at 1 mmol Eu^3+^ ion concentration, and then was quenched at higher concentrations. The concentration quenching effect occurs due to the decrease in Eu-Eu distance, which causes the non-radiative energy transfer between the activator ions. The variation in the emission intensity with activator ion concentration is shown in Fig. 5(c).

**Figure 6:**
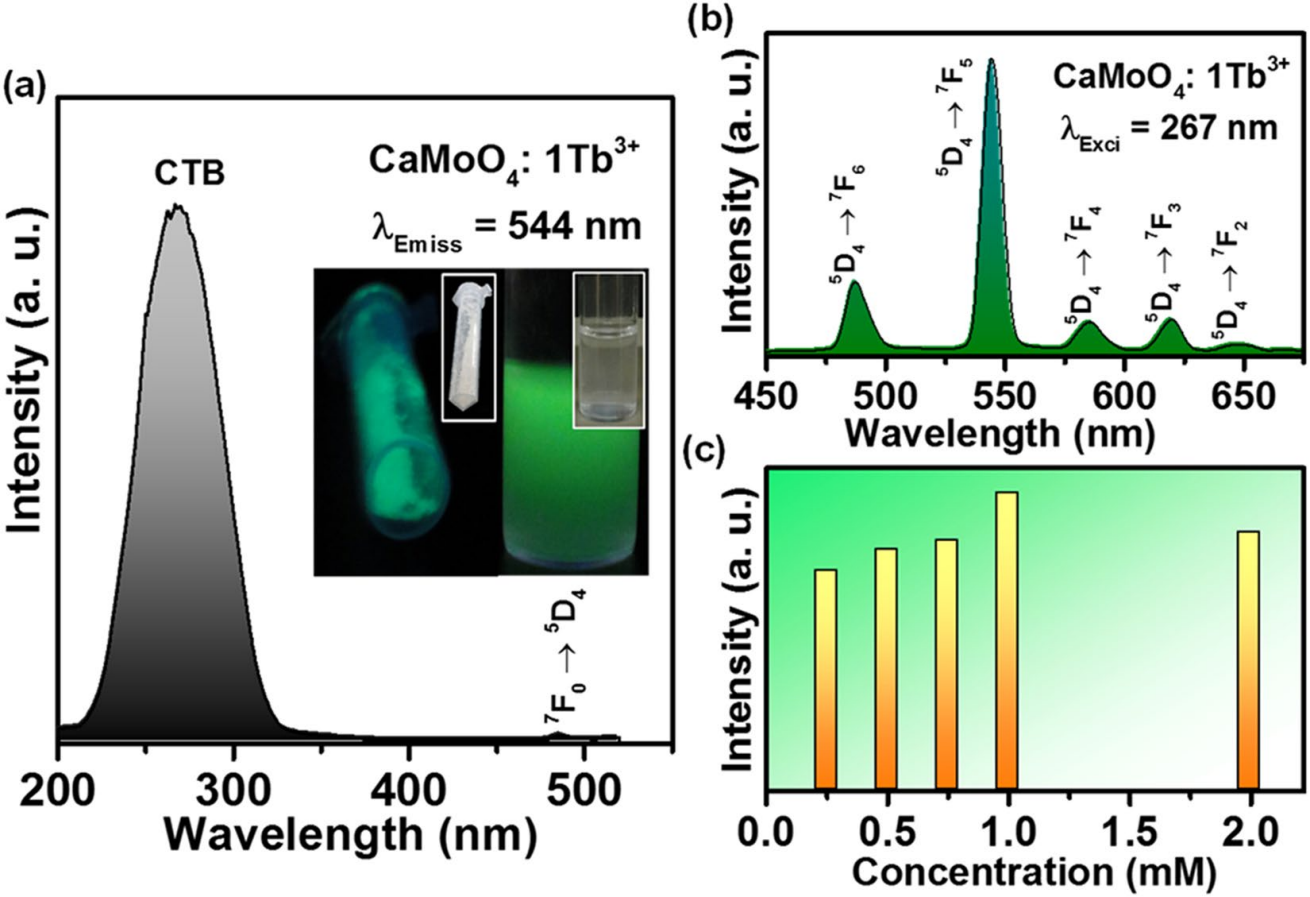
(**a**) PLE spectrum (inset shows the photographs of the powder sample and the powder dispersed in DI water under normal and UV lights), (**b**) PL emission spectrum and (**c**) PL emission intensity versus Tb^3+^ ion concentration for the Tb^3+^ ions doped CaMoO_4_ sample.

Figure 6 shows the PLE and PL emission properties of the Tb^3+^ ions doped CaMoO_4_ sample. As shown in Fig. 6(a), the PLE spectrum in the wavelength range of 200–550 nm was observed under the emission wavelength of 544 nm. The excitation spectrum consisted of CTB in the UV region which is due to the charge transfer between the O^2−^ and Mo^6+^ ions and f-f transitions of Tb^3+^ ions in the longer wavelength region. The PL emission spectrum in the wavelength range of 450–675 nm was observed under the UV excitation of 267 nm (Fig. 6(b)). The emission spectrum shows the bands due to the transition from the higher energy ^5^D_4_ state to the ground state ^7^F_j_ (j = 2, 3, 4, 5 and 6) levels. Among all the emission bands, the PL emission intensity of the ^5^D_4_ → ^7^F_5_ electronic transition is high, which gives the characteristic green emission. The emission colors from the powder sample and the powder dispersed in DI water were snapped under normal and UV lights and are shown in the inset of Fig. 6(a). The PL emission intensity increased with the increase of dopant concentration and reached its maximum for 1 mmol of Tb^3+^ ion concentration. The emission intensity decreased further with the increase in the concentration of activator ions, which is due to the concentration quenching effect. The quenching in emission intensity is due to the shrinkage of Tb-Tb distance which causes the increase in the energy transfer to non-radiative sinks. The change in the emission intensity with respect to the activator ion concentration is shown in Fig. 6(c).

**Figure 7:**
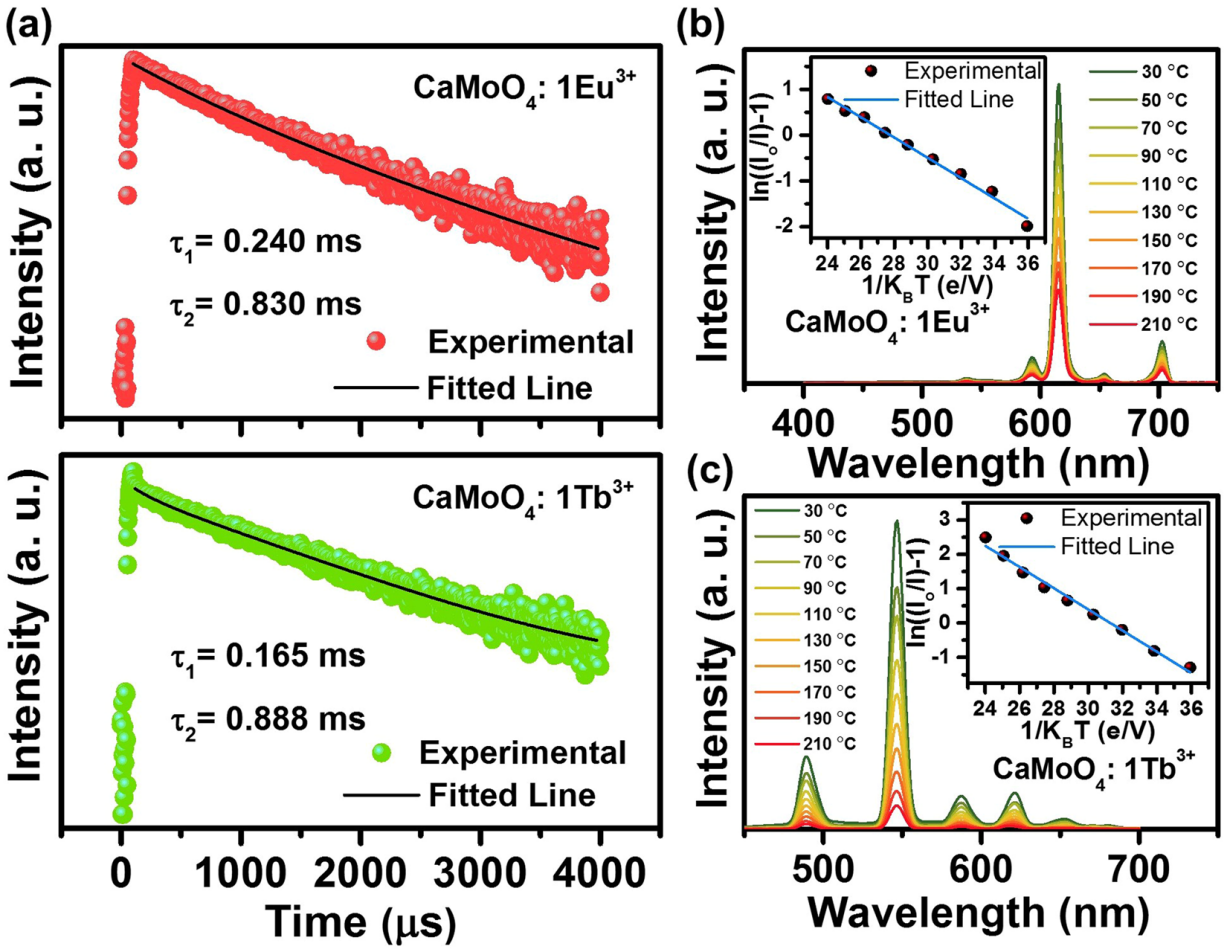
(**a**) Luminescence decay curves and (**b**,**c**) temperature-dependent PL emission spectra of the Eu^3+^ and Tb^3+^ ions doped CaMoO_4_ samples. Inset of (**b**,**c**) shows the linear fitting results for ln((I_o_/I)−1) versus 1/K_B_T.

The luminescence decay curves of the Eu^3+^ and Tb^3+^ ions doped CaMoO_4_ samples were recorded as shown in Fig. 7(a). The decay curves were measured at an excitation wavelength of 267 nm and emission wavelengths of 614 and 544 nm for Eu^3+^ and Tb^3+^ ions doped CaMoO_4_ samples, respectively. The decay curves were well fitted to the double exponential decay function as shown below:

4 $${\rm{I}}({\rm{t}})={{\rm{I}}}_{{\rm{o}}}+{\rm{Ae}}\frac{-t}{{\tau }_{1}}+{\rm{Be}}\frac{-t}{{\tau }_{2}},$$

where I(t) and I_o_ are the intensities at time t and 0. τ_1_ and τ_2_ are lifetime values and A and B are constants. The existence of double exponential behaviour is due to the presence of energy transfer from host lattice to activator ions and/or due to the presence of oxygen vacancies^29, 30^. The presence of oxygen vacancies in the Eu^3+^ and Tb^3+^ ions doped CaMoO_4_ samples was proved from the XPS results. The thermal stability is also an important parameter to be considered when it comes to the rare-earth doped phosphor materials. The temperature-dependent PL emission spectra of the Eu^3+^ and Tb^3+^ ions doped CaMoO_4_ samples in the wavelength rage of 400–700 nm are shown in Fig. 7(c) and (d). The emission intensity decreased gradually with the increase of temperature from 30 to 210 °C (step size of 20 °C). Generally, when the temperature is increased, the phonon manifold occurs in the high vibration levels and then is quenched to the ground states non-radiatively via the crossover between the ground and excited states. Hence, a drop in the emission intensity was observed with the increase of temperature. Additionally, the temperature at which the emission intensity drops to 50% of its original value called as thermal quenching temperature (T_0.5_) was also observed. The normalized intensity versus temperature was plotted as shown in Fig. S3. The T_0.5_ values for the Eu^3+^ and Tb^3+^ ions doped CaMoO_4_ samples were found to be 146 and 99 °C, respectively. The activation energy (E_a_) values for the Eu^3+^ and Tb^3+^ ions doped CaMoO_4_ phosphor powders were obtained from the plots drawn between ln ((I_o_/I)−1) versus 1/K_B_T^31^. The plots were then fitted linearly as shown in the inset of Fig. 7(c) and (d). The slope of the straight line fitting gives the E_a_ values and the values were found to be 0.222 and 0.308 eV for the Eu^3+^ and Tb^3+^ ions doped CaMoO_4_ phosphor powders, respectively.

**Figure 8:**
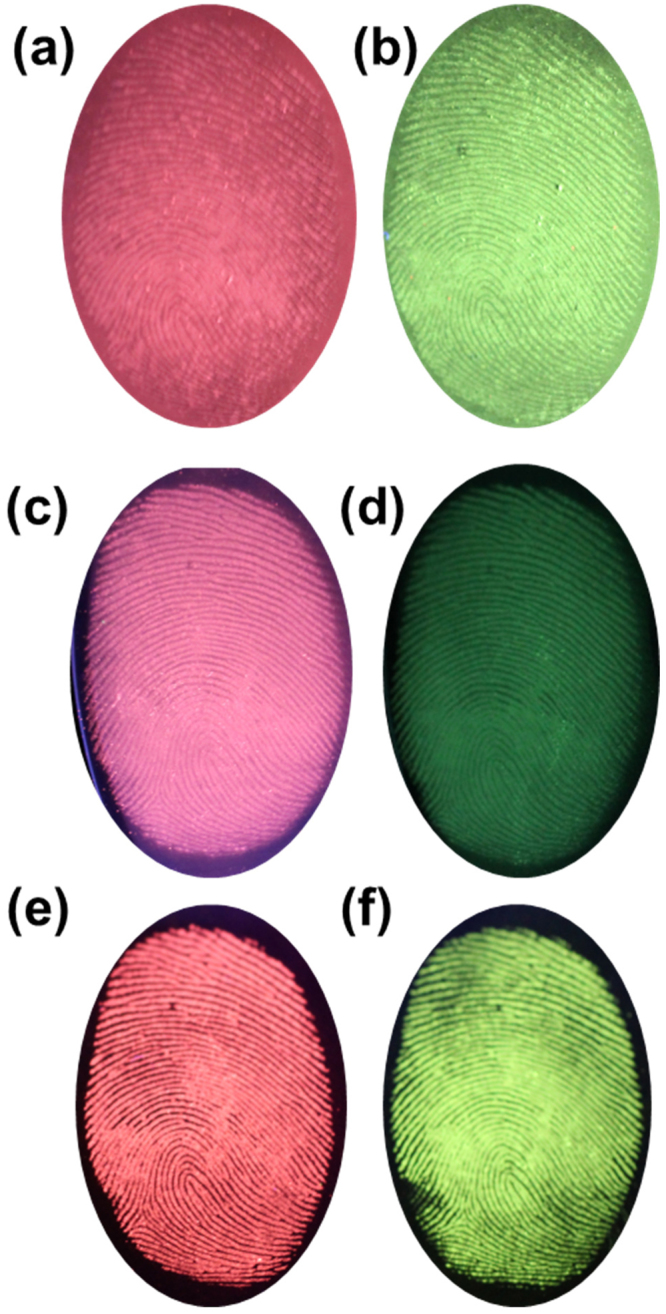
Digital photographs of the visualized CaMoO_4_:Eu^3+^ and CaMoO_4_:Tb^3+^ spheres stained latent fingerprint images on different substrates (**a**,**b**) slide glass, (**b**,**c**) CD and (**e**,**f**) stainless steel cup.

The luminescence properties of CaMoO_4_ nanomaterials doped with Eu^3+^ and Tb^3+^ ions have been studied for being used in latent fingerprint detection. The size of these nanomaterials was useful for effective detection of minute details in the fingerprints. The strong visible light emissions (red and green) from these nanomaterials under UV (254 nm) light were also useful for latent fingerprint detection with low background interference and high contrast. Figure 8 shows the fingerprint images developed by Eu^3+^ and Tb^3+^ ions doped CaMoO_4_ phosphors on different substrates i.e., slide glass (Fig. 8(a) and (b)), compact disc (CD) (Fig. 8(c) and (d)) and stainless steel cup (Fig. 8(e) and (f)). The clear and bright images under the UV light indicate that the latent fingerprints on different kinds of substrates can be smoothly detected using these phosphor materials.

**Figure 9:**
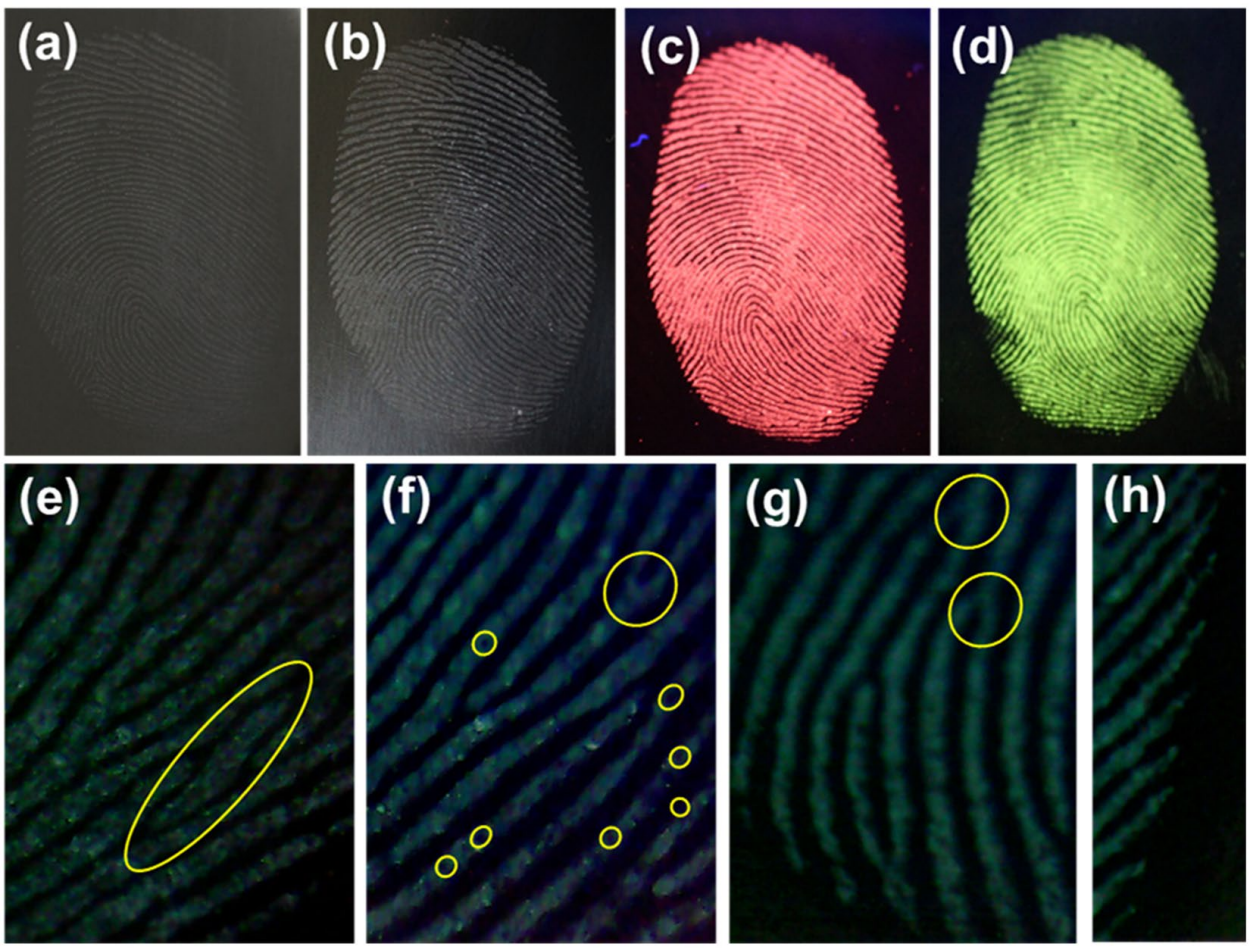
(**a**) Bare image, (**b**) powdered image, (**c**,**d**) fluorescent images and (**e**–**f**) high-magnification images of the latent fingerprints under UV light irradiation, which were obtained from the surface of stainless steel cup.

**Figure 10:**
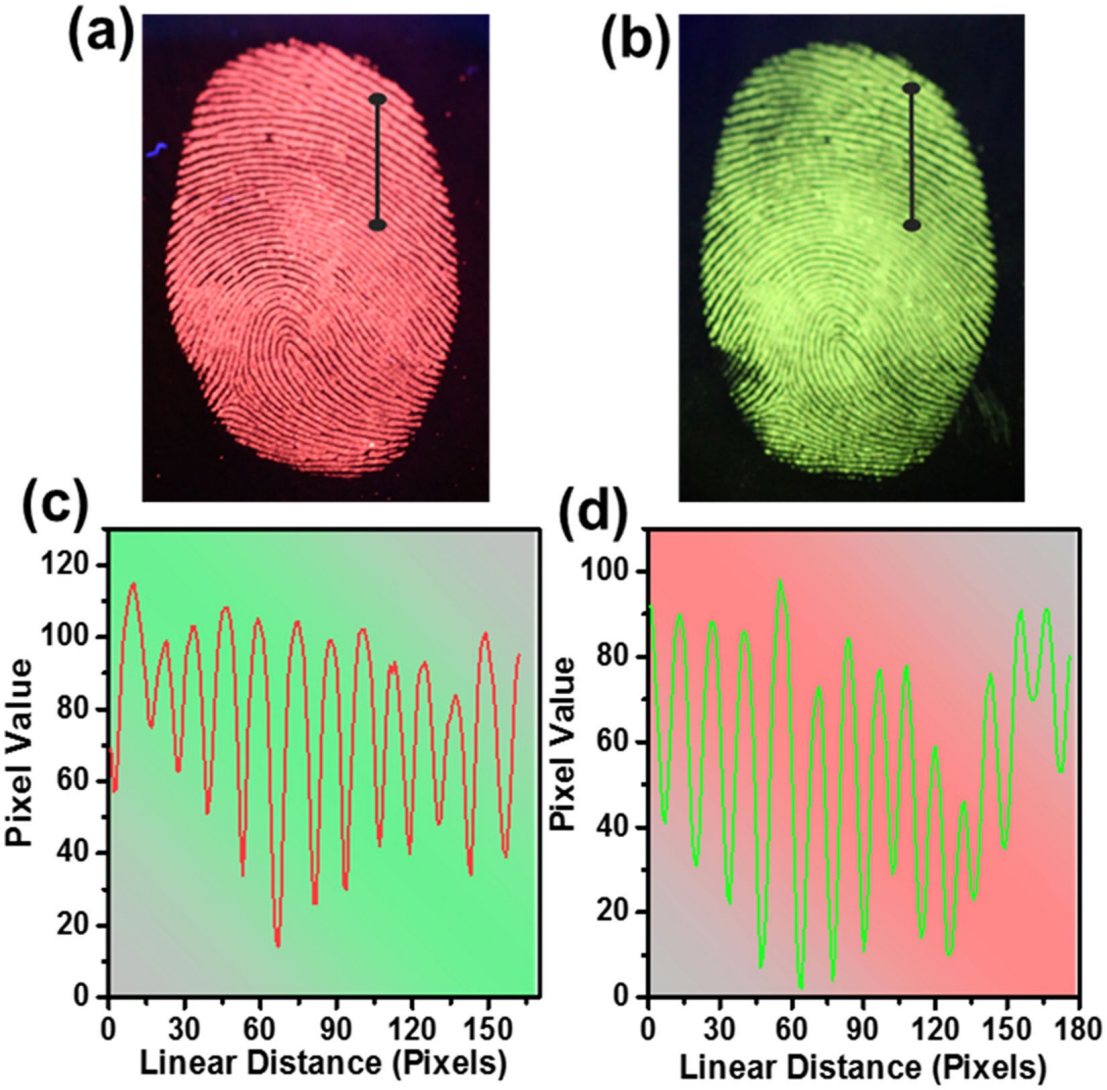
(**a**,**b**) Area highlighted with solid line on the digital photographs of the visualized latent fingerprint images to verify the affinity of the rare-earth ions doped CaMoO_4_ spheres and (**c**,**d**) corresponding fluctuations in red and green values and the difference between ridges and furrows over the highlighted papillary ridges.

**Figure 11:**
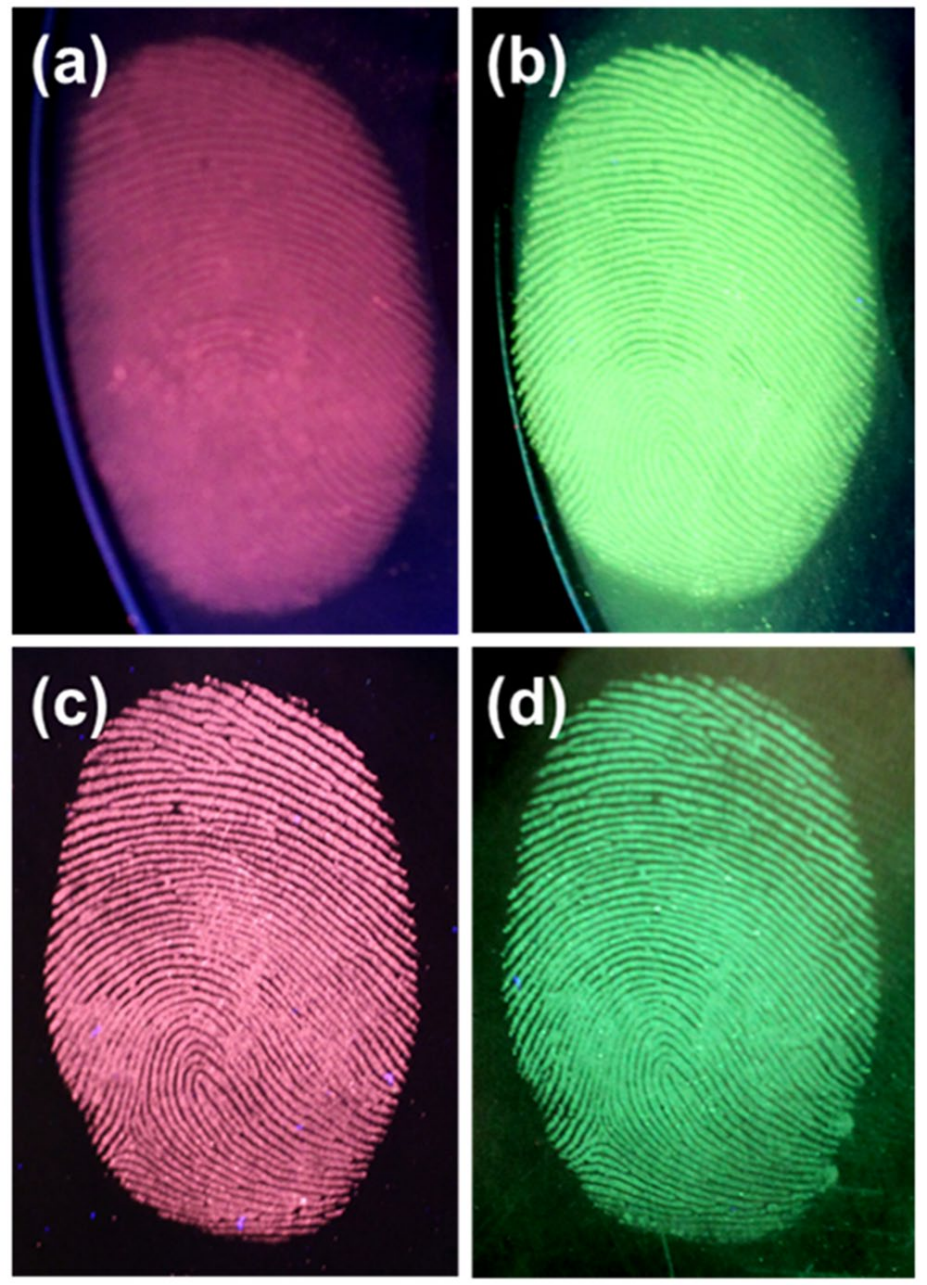
Digital photographs for the visualization of latent fingerprints on (**a**,**b**) CD and (**c**,**d**) stainless steel cup, which were stained with CaMoO_4_:Eu^3+^ and CaMoO_4_:Tb^3+^ spheres after 9 days of aging in ambient atmosphere.

The latent fingerprint is one of the most common evidences found in any crime scenes and it is the one which presents the problem of invisibility. Therefore, application of chemical, physical or optical treatments is required to see the latent fingerprint^32^. Herein, we used the physical technique, i.e., powdering for the detection of latent fingerprints. To get into the details of the latent fingerprint, a specified substrate was selected, i.e., stainless steel cup. The detailed procedure of physical technique for developing the latent fingerprint was described in experimental section. The fresh fingerprint image and the powdered image are shown in Fig. 9(a) and (b). The latent fingerprint image shown in Fig. 9(b) is noticeable due to the attraction and adhesion of particles to the moisture and oily components present in the latent fingerprint. The fluorescent images under UV light showing the red and green emission colors are shown in Fig. 9(c) and (d). The fingerprint of one person is different from others and it is unique entity for the identification of a person. The fingerprint contains many minute details like bifurcations, islands, enclosures, etc. which are called as Galton details. The number and position of these minute details vary from person to person and are very important for individualization. The minutiae found in green color latent fingerprint image are shown from Fig. 9(e)-(h). The island found in the fingerprint is shown in Fig. 9(e) and the bifurcations and sweat pores found are shown in Fig. 9(f) and (g). All these details were marked with yellow circles in the figures. The ending of ridges are shown in Fig. 9(h) and displaying these minute details evidently makes these nanomaterials a potential candidate for latent fingerprint detection. The high-magnification optical images from Fig. 9(e)-(h) also revealed that the powder was attached only to the ridges, and the grooves were empty and do not show any emission color. To further confirm this, we have taken the pixel profile for both red and green optical latent fingerprint images. The pixel profile was obtained for a small area which was highlighted with a solid line on the digital photographs of the visualized latent fingerprint images (Fig. 10(a) and (b)). The corresponding fluctuations in red and green pixel values and the difference between ridges and furrows of the highlighted papillary ridges are shown in Fig. 10(c) and (d). From these pixel profile data, it is further confirmed that the grooves are empty and the powder was attached only on the ridges of the fingerprint. To test the quality of the powder and its affinity characteristics, the fingerprints taken on CD and stainless steel cup are preserved in ambient atmosphere for 9 days. Later, the latent fingerprint fluorescent images were observed under UV light as shown in Fig. 11. The imagesin Fig. 11(a) and (b) are the latent fingerprints on the CD and the images in Fig. 11(c) and (d) are the latent fingerprints on the stainless steel cup. As mentioned earlier, the affinity of the powder is due to the presence of moisture and oily components in the latent fingerprint. Actually, the finger ridges are appended only to the eccrine sweat glands. These glands are found all over the body and functions as thermoregulators. The secretions from these glands have 99% of water and the remaining 1% constituents include inorganic salts and organic derivatives. The derivatives include amino acids, proteins, lactic acid, urea, sugars, uric acid, creatinine and choline^33^. After a period of time, the water evaporated from the surface of the fingerprint and the oily components remained. Of these oily components, the amino acids remained as solid materials on the surface of the fingerprint and there are reports saying that the detectable amount of amino acids is found in fingerprints. The oxide materials react with the traceable amount of amino acids present in the latent fingerprint mark and have strong affinity. Hence, the finger marks were clearly observed even after 9 days of aging in ambient atmosphere.

## Conclusion

In summary, the synthesis of spherical-shaped CaMoO_4_ nanoparticles using the mixture of IPA and DI water was studied. The samples were crystallized in tetrahedron phase with a space group I4_1_/a (88) and the XRD patterns of the Eu^3+^ and Tb^3+^ ions doped samples well matched with that of the as-prepared sample along with the standard JCPDS card value. The luminescent properties of the Eu^3+^ and Tb^3+^ ions doped samples exhibited strong red and green visible emissions under UV irradiation. These materials were then used as an effective material for the detection and enhancement of latent fingerprints on different surfaces, including slide glass, compact disk and stainless steel cup. The fingerprint images were clear with high contrast and low background interference, and even showed the minute details which help for individualization. The affinity of the powders to the fingerprints was good, which results in an evident fingerprint ever after 9 days of aging in ambient atmosphere. The above mentioned results manifest the characteristics of CaMoO_4_ phosphor materials and the versatile nature as fluorescent powders for latent fingerprint detection.

## Experimental Procedure

### Materials

Calcium nitrate tetrahydrate (Ca(NO_3_)_2_∙4H_2_O), ammonium heptamolybdate tetrahydrate ((NH_4_)_6_Mo_7_O_24_∙4H_2_O), sodium hydroxide (NaOH), europium nitrate pentahydrate (Eu(NO_3_)_3_∙5H_2_O), terbium nitrate hexahydrate (Tb(NO_3_)_3_∙6H_2_O) and ethylene diamine tetraacetic acid (EDTA) were employed. All the chemicals are of analytical grade, purchased from Sigma-Aldrich Co. and are used as received without any further purification. Isopropyl alcohol (IPA) was purchased from ChemiTop Co., South Korea and the de-ionized (DI) water was obtained from a Milli-Q synthesis system (resistivity of 18.2 MΩ-cm).

### Synthesis

For preparing the CaMoO_4_ spherical particles, three solutions, namely, solution-I, -II and -III were prepared as follows. Solution-I was prepared by mixing 35 mmol of calcium nitrate tetrahydrate in 70 ml of IPA, and 0.2 M 10 ml sodium hydroxide solution was prepared and labeled as solution-II. Solution-III was prepared by mixing 5 mmol of ammonium heptamolybdate tetrahydrate in 20 ml of DI. All the solutions were stirred to form transparent solutions. After few minutes, the solution-II was added dropwise to the solution-I. Later, the solution-III was added to the above mixture under continuous stirring. Finally, 0.1 g of EDTA was added and the solution was transferred to the teflon-lined stainless steel autoclave and heated to 160 °C for 5 h. The autoclave was further cooled down to room temperature, and the powder was collected, washed with DI water and dried at 70 °C. The europium and terbium doped samples were prepared by following the similar experimental procedure except adding stoichiometric amounts of europium nitrate pentahydrate/terbium nitrate hexahydrate to the solution-I.

### Characterization

The prepared powders were characterized by using the following instruments or techniques. Field-emission scanning electron microscope (FE-SEM: LEO SUPRA 55, Carl Zeiss) equipped with energy dispersive X-ray spectrometer (EDX), X-ray diffractometer (XRD: M18XHF-SRA, Mac Science), Fourier transform infrared spectrometer (FTIR: Spectrum 100, PerkinElmer), X-ray photoelectron spectrometer (XPS: Thermo Electron Multilab2000) and spectroflourometer (FluroMate FS-2, Scinco, South Korea) equipped with a temperature controlled heating holder (25–250 °C) were employed.

### Latent fingerprint detection

Substrates like slide glass, compact disk and stainless steel cup were selected for detecting the latent fingerprints. Fresh fingerprints were obtained by adopting the following procedure; the fingers were cleaned with soap and water and dried by exposing to air. Later, the finger was mildly rubbed over the forehead and pressed against different substrates as mentioned above. For studying the aging effect on fingerprints, the latent fingerprints on preferred substrates were preserved for 9 days in ambient atmosphere. Subsequently, the prepared fluorescent nanomaterials were brushed on to the surface of the substrates carefully. The excess powder was removed from the substrates carefully by smooth and mild motion for getting the latent fingerprint images. The latent fingerprints on different substrates under 254 nm UV light illumination were photographed with a Nikon D300 digital camera equipped with a Nikon AF-S VR 55 mm f/2.8 G IF-ED Macro lens without any filters.

### Statement of authors and informed consent

The authors confirmed that all experiments (taking fingerprints of a volunteer/individual) were performed in accordance with relevant guidelines and regulations. An explicit informed consent was obtained from the anonymous volunteer providing the fingerprints. The individual explicitly allowed the authors to use the data in the present publication.

## Electronic supplementary material

Supplementary Info
