# Supplementary material for: Red and green colors emitting spherical-shaped calcium molybdate nanophosphors for enhanced latent fingerprint detection
Source: Sci Rep. 2017 Sep 14;7:11571. doi: 10.1038/s41598-017-11692-1 (PMC5599674; doi:10.1038/s41598-017-11692-1)
Supplement: Supplementary file 1 — Supplementary Info [file 41598_2017_11692_MOESM1_ESM.pdf]

Supporting Information

Red and green colors emitting spherical-shaped calcium molybdate nanophosphors for enhanced latent fingerprint detection

L. Krishna Bharat[1], G. Seeta Rama Raju[2], and Jae Su Yu[1]*

[1] Department of Electronic Engineering, Institute for Wearable Convergence Electronics, Kyung Hee University, Yongin-si, Gyeonggi-do 17104, Republic of Korea
[2] Department of Energy and Materials Engineering, Dongguk University-Seoul, Seoul 04620, Republic of Korea

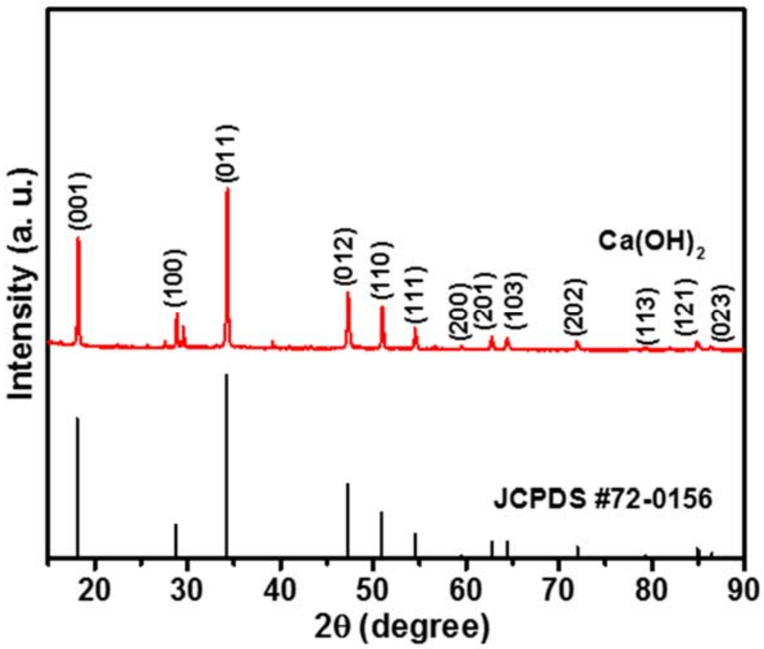

Figure S1. XRD pattern of $Ca(OH)_2$.

*Address correspondence to jsyu@khu.ac.kr

Tel: +82-31-201-3820; Fax: +82-31-206-2820

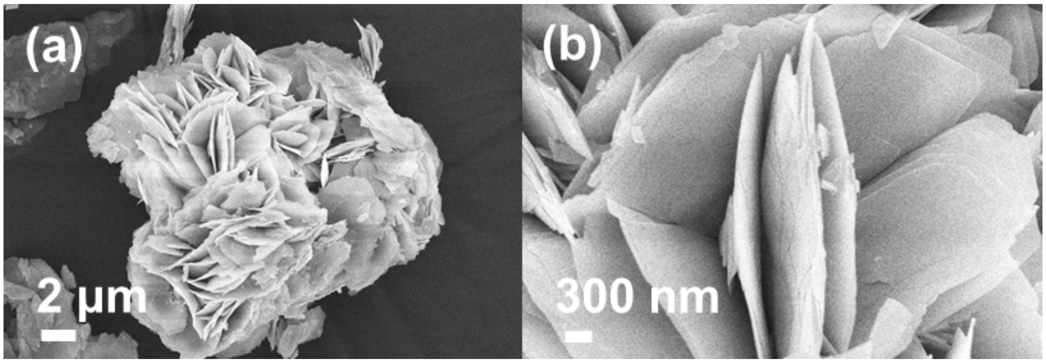

Figure S2. (a) Low-magnification and (b) high-magnification FE-SEM images of the $CaMoO_4$ prepared in pure DI water.

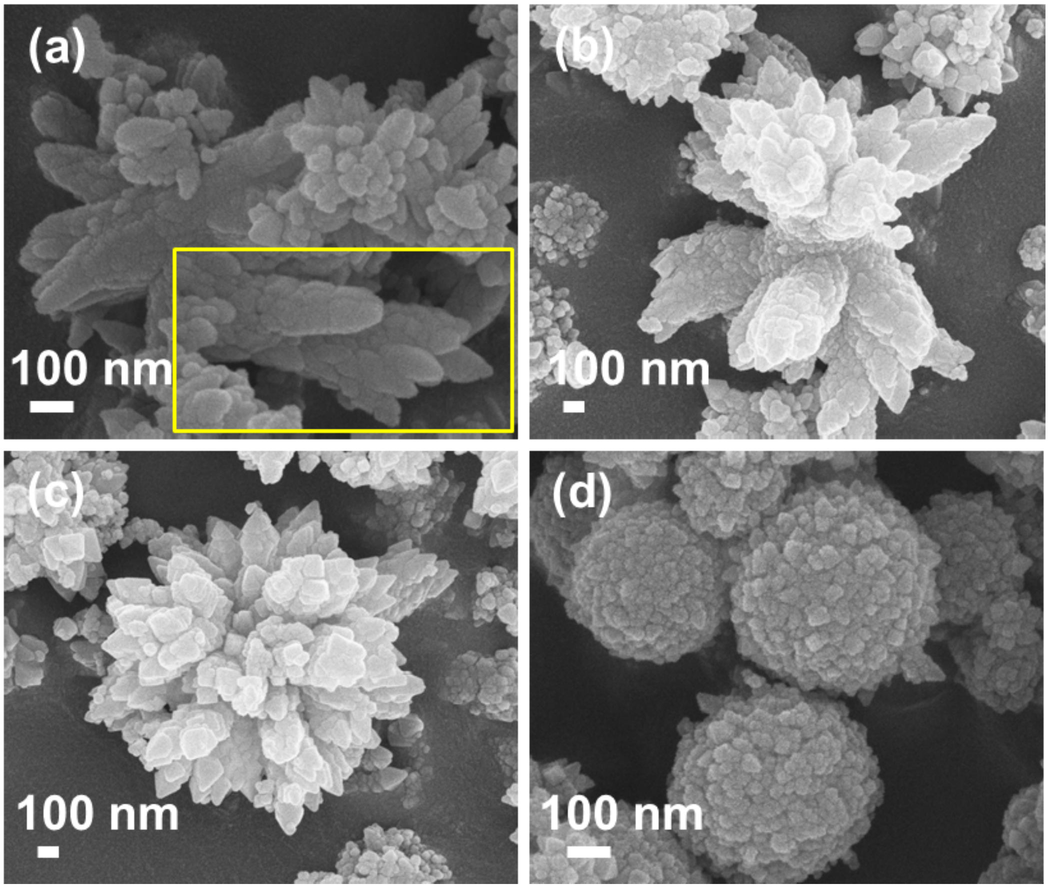

Figure S3. (a-d) FE-SEM images showing the growth mechanism.

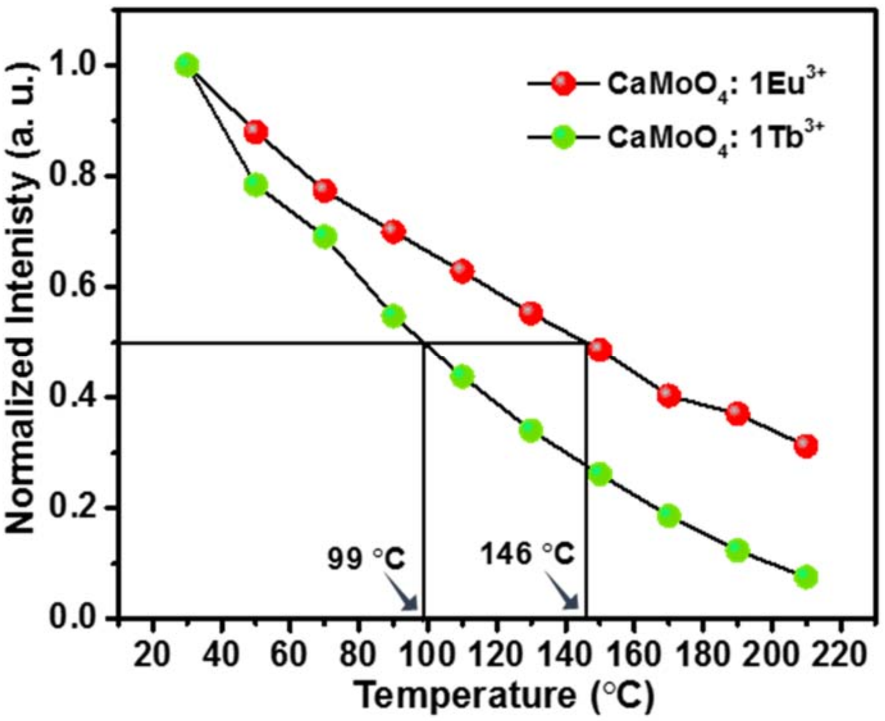

Figure S4. Temperature-dependent PL emission intensities for the Eu^{3+} and Tb^{3+} ions doped $CaMoO_4$ samples.
